# Retraction: Comprehensive computational analysis reveals H5N1 influenza virus-encoded miRNAs and host-specific targets associated with antiviral immune responses and protein binding

**DOI:** 10.1371/journal.pone.0325170

**Published:** 2025-05-23

**Authors:** The PLOS One Editors

The *PLOS One* Editors retract this article [1] because it was identified as one of a series of submissions for which we have concerns about potential manipulation of the publication process. These concerns call into question the validity and provenance of the reported results. We regret that the issues were not identified prior to the article’s publication.

FN, MHS, and MRJ did not agree with the retraction. JTC responded but expressed neither agreement nor disagreement with the editorial decision. UAA, MKO, MAAM, YAA, WHAQ, HA, GY, and SA either did not respond directly or could not be reached.
